# Call for Action to Address Equity and Justice Divide During COVID-19

**DOI:** 10.3389/fpsyt.2020.559905

**Published:** 2020-12-03

**Authors:** Sonu Bhaskar, Aarushi Rastogi, Koravangattu Valsraj Menon, Beena Kunheri, Sindhu Balakrishnan, Jeremy Howick

**Affiliations:** ^1^Pandemic Health System REsilience PROGRAM (REPROGRAM) Consortium, REPROGRAM Health Equity and Justice Study Group, Sydney, NSW, Australia; ^2^Department of Neurology, Liverpool Hospital and South Western Sydney Local Health District, Sydney, NSW, Australia; ^3^Neurovascular Imaging Laboratory & NSW Brain Clot Bank, Ingham Institute for Applied Medical Research, The University of New South Wales, Sydney, NSW, Australia; ^4^South West Sydney Clinical School, The University of New South Wales Sydney, Sydney, NSW, Australia; ^5^Department of Psychiatry, South London and Maudsley NHS Foundation Trust, Kings Health Partners, London, United Kingdom; ^6^Manasvi, Kochi, India; ^7^Department of Radiation Oncology, Amrita Institute of Medical Sciences, Amrita Vishwa Vidyapeetham, Kochi, India; ^8^Department of Anaesthesia and Critical Care, Amrita Institute of Medical Sciences, Amrita Vishwa Vidyapeetham, Kochi, India; ^9^Faculty of Philosophy and Oxford Empathy Programme, University of Oxford, Oxford, United Kingdom

**Keywords:** social determinants of health, COVID-19, vulnerable communities, mental health, health policy, health equity, social medicine, social justice

## Abstract

The coronavirus 2019 disease (COVID-19) is deepening the inequity and injustice among the vulnerable communities. The current study aims to present an overview of the impact of COVID-19 on equity and social justice with a focus on vulnerable communities. Vulnerable communities include, but not limited to, healthcare workers, those from lower socioeconomic backgrounds, ethnic or minority groups, immigrants or refugees, justice-involved populations, and people suffering from chronic diseases or mental illness. The implications of COVID-19 on these communities and systemic disparities beyond the current pandemic are also discussed. People from vulnerable communities' experience disproportionately adverse impacts of COVID-19. COVID-19 has exacerbated systemic disparities and its long-term negative impact on these populations foretell an impending crisis that could prevail beyond the COVID-19 era. It is onerous that systemic issues be addressed and efforts to build inclusive and sustainable societies be pursued to ensure the provision of universal healthcare and justice for all. Without these reinforcements, we would not only compromise the vulnerable communities but also severely limit our preparedness and response to a future pandemic.

## Introduction

Outbreaks such as the coronavirus 2019 (COVID-19) challenge our existing health and justice systems (1–5). The health systems around the world have been repurposed to contain and mitigate the COVID-19 infection rate and provide acute care to COVID-19 patients requiring hospitalization (6). Furthermore, due to quarantine measures physical access to health and justice systems have been limited for those with ongoing and emergent needs. These systems have been forced to adapt and reconfigure (7, 8), with disproportionate implications on vulnerable populations (3, 9). For example, COVID-19 accelerated rapid adoption and expansion of telemedicine (10), and repurposing of existing clinical wards to provide COVID-19 clinical service (8, 11). In these unprecedented times, issues related to equity and justice must be considered (12, 13). Lack of these considerations will put those from vulnerable communities at harm (14). The health, psychological, social and economic dimensions of an individual determine the opportunity, or the lack of it, to health and justice and the underpinning principles of equity, fairness and inclusiveness. Ensuring that the various risks to the vulnerable populations are identified early and appropriate measures are taken to prevent their impact. This article sought to present an overview of the impact of COVID-19 on vulnerable populations with regards to the issues of health equity and justice. We also provide targeted recommendations and call for a concerted action to address acute and system disparities in health equity and justice.

## Methods

A scoping review on PubMed/Medline, media sources and official government websites were performed using the keywords “Equity,” “Justice,” and “COVID-19” until April 18, 2020, at the time of writing this manuscript. A Population, Intervention, Comparison, and Outcomes (PICO) search strategy was used (15), with the general population and healthcare workers as the study population, COVID-19 as the intervention, status of health equity and justice before COVID-19 as the comparison arm and impact of COVID-19 on equity and justice in the study population as the outcomes. Some recent publications were considered during the revision of the manuscript during October 2020 following a rather long review. The impact of COVID-19 on vulnerable populations including healthcare workers, people from lower socioeconomic backgrounds, ethnic or minority groups, immigrants or refugees, justice-involved populations, and people suffering from chronic diseases or mental illness were studied. Appropriate references relevant to COVID-19 vis a vis equity and justice issues were included in the final synthesis. Besides, we also provide targeted recommendations to address acute and systemic inequity and injustice issues during and beyond COVID-19.

## Results

### Impact of COVID-19 on Healthcare Workers

Healthcare workers are disproportionately at higher risk in COVID-19 in comparison with the general community (16, 17). Extended exposure to large numbers of infected patients places them at direct risk of contracting the infection. This is exacerbated by the lack of personal protective equipment (PPE), which has been a subject of major concern across the world (18, 19). Reports of an increasing number of healthcare worker deaths due to COVID-19 have created a sense of fear and outrage (20–22). Workers have also reported anxiety about transmitting the infection to their families, elderly parents and young children (23, 24). There is a critical need for increased efforts to provide adequate PPE (18).

Healthcare workers are also suffering an immense psychological strain (22, 25–27), having to make difficult triage decisions and witnessing the loss of several patients and colleagues (28). Moreover, workers with young children are likely to be dramatically impacted by school closures (23). To protect this population from extreme physical and mental exhaustion, governments must recognize their need for rest, and should also consider practical measures to provide support, such as the provision of food and care for young children. Targeted resources should be made available to enhance the mental health of healthcare workers. Healthcare workers, like the general population, also carry the burden of certain chronic conditions, which may put them at increased risk of COVID-19 infection (29). Considerations on age, underlying comorbidities and mental health of healthcare workers must be taken while rostering for frontline COVID-19 related care or repurposing (30, 31). Many healthcare workers may have to quarantine themselves to limit the risks to their families. It is also disheartening to note the stories of stigmatization of healthcare professionals in some parts of the world by refusing accommodation and targeting health care workers due to lack of concern and understanding.

There have been reports on disproportionately high rates of COVID-19 related death in medics from black and minority ethnic (BAME) backgrounds in the United Kingdom (UK) (32–34) and this has resurfaced the ongoing debate in the UK that those from BAME backgrounds are often not given adequate support from their peers and often struggle to be treated equally by peers (33, 35, 36). In addition to bullying and systemic racism (37), concerns about disproportionate representation in the senior management and decision-making teams or boards have also been reported by the BAME healthcare workers (38). The medical and public health administration must ensure that all clinicians, especially those from minority ethnic backgrounds, are provided adequate sponsorship and support including peer mentorship (39). Furthermore, COVID-19 has also impacted traditional medical education and training (40). Use of technologies in delivering medical education remotely could minimize the impact on medical students and medical education, alike.

### Impact of COVID-19 on Lower Socioeconomic Communities

Data shows that low socioeconomic communities are bearing a disproportionately higher brunt of the pandemic (41–45). These groups face a greater risk of detrimental financial and health outcomes during the crisis. Low-income workers in industries such as retail, transport and labor are unable to work from home and thus risk losing their source of income should they discontinue their on-site work to protect themselves and their families from COVID-19 exposure (46). Such interruptions in income can disrupt food security for families, and also limit their ability to afford treatment of existing health conditions. People working in essential sectors (e.g., transport, postal services), however, face an increased risk of contracting COVID-19 infection as they must continue working. Along with the increased risk of infection, they may also have greater difficulty in following evidence-based guidance like social distancing, accessing COVID-19 testing and care facilities. We implore governments and essential sector organizations to provide financial support, a safe working environment to support the safe functioning of essential services during the pandemic. The government should consider looking at employment opportunities to enable lower socioeconomic groups to continue their livelihood. Moreover, there should be an increase in the access to and provision of free testing to people from low socioeconomic backgrounds, and deployment of mobile screening and infectious disease monitoring facilities in these communities.

Low socioeconomic groups are also at greater health risks due to unfavorable living conditions. Homeless people, or those living in overcrowded housing, are unable to adhere to social distancing guidelines and often lack access to personal hygiene facilities (47). Given the increase in the incidents of domestic violence, it is unsafe for them to adhere to lockdown laws and stay at home (48). We recommend the provision of temporary accommodation for people facing financial distress, homelessness, overcrowding or domestic abuse. Governments should also ensure essential supplies of electricity, water and sanitation be maintained. Furthermore, adequate access to the internet and technology should be provided to enable the continuity of education to children from lower socioeconomic households. Girls and women are more likely to be disproportionately impacted due to the closure of schools (49, 50). Increase in sexual exploitation, pregnancy and forced marriage due to closure of schools and a prolonged period of home quarantine would lead to higher drop-out rates among teenage girls (50). Furthermore, a disproportionate increase in unpaid household work burden on girls may limit their study time and hence negatively affect their academic performance and progression—causing an increase in school drop-outs (50). The government must work closely with grass-root non-government organizations to inform and educate people about the importance of continuity of education. Universal basic income could be considered to minimize the emergent effects of COVID-19 (51).

People from these communities also tend to have poor levels of education and literacy, and as a result, do not adequately receive public health messages (47). Adherence to public health recommendations is essential to reducing exposure to infection. Thus, there must be an increase in targeted efforts to improve awareness about public health measures such as social distancing, regular handwashing and use of masks among people from low socioeconomic backgrounds. Therefore, public health initiatives should aim to actively identify such communities and adapt suitable medium of communication utilizing community-level health workers.

### Impact of COVID-19 on Ethnic or Minority Groups

Pandemics invoke irrational fear and uncertainty (52). These are propitious grounds for the vagaries of hate, stigma, discrimination, racism and xenophobia to develop. Unfortunately, people from ethnicity/minority groups such as blacks, people from minority groups e.g., those from Asian and Indigenous backgrounds are more likely to be the target of hate, abuse, and sometimes violence (53). The act of xenophobia takes various channels including social media. There are increasing reports of people from Asian backgrounds being targeted (54). It is a valid argument to postulate that these acts can rapidly scale through misinformation and social media and messaging applications, warranting a need for governments to continually monitor such events or sentiments. Targeted awareness programs are required by appropriate authorities to debunk the myth linking specific communities to COVID-19. Efforts to reinforce that pandemics such as COVID-19 affect one and all, irrespective of culture, community, creed, sex, race, and ethnicity. Infectious disease is agnostic of ethnicity, race, and cultural background of the people it affects. Political leadership must observe caution and desist from making inflammatory statements that could invoke stigmatization and xenophobia.

In the United Kingdom (UK), increasing concerns have been raised over the disproportionately higher proportion of deaths of people from the black and minority ethnic (BAME) background and those working for the National Health Service (NHS) during the COVID-19 (1). The UK government has launched an official review into why members of the BAME community are worst hit.

Previous data have shown that people of ethnicity and color have relatively poor access to healthcare services and the provision of treatment (55–58). During crises like COVID-19, these syndemic factors become important as to how historical health and social disparities along with emerging or current factors, such as epidemics, exacerbate the negative consequences on ethnic or minority groups (58). Therefore, authorities should ensure that pharmacies or local primary healthcare facilities are accessible to these communities in hours of need. Special consideration with regards to public health measures needs to be taken to protect and safeguard the health and well-being of people from indigenous backgrounds. One such measure taken by the Australian government was the limiting of travel to areas inhabited by indigenous populations in the Northern Territory, to isolate and protect these communities from exposure to COVID-19 (59).

Indigenous communities face a significant lack of access to health and justice systems (60, 61). Justice is critical to ensure that the individual rights of these communities are protected in these uncertain times. In a crisis like COVID-19, these communities are more likely to face a greater burden of unemployment, which may have a potentially cascading impact on their families. These considerations need to be addressed by the concerned family and welfare government institutions. Any public health preventative interventions or measures should be developed in consultation and with the informed consent of Indigenous people.

### Impact of COVID-19 on Immigrants and/or Refugees

Recent years have witnessed a meteoric rise in the mass-scale forced displacement of people due to climate change, political crisis, humanitarian disaster, war and violence. Tertiary care health systems remain the safety net to these vulnerable populations who have fled homes without a certain abode. Migrants and refugees are at a higher risk of infection and negative consequences of pandemics due to desperately poor sanitation facilities, cramped conditions, limited access to healthcare and lack of financial resources to sustain families (62, 63). In places such as Kutupalong Camp in Bangladesh, currently housing over 600,000 displaced people in a mere area of 13 square kilometers, practicing social distancing is near impossible (64). Without access to clean water, promotion of hand-washing guidelines is also of little use in such camps. Should an outbreak occur in these areas, it is likely to spiral out of proportion. Authorities must make serious efforts toward providing clean water and sanitation facilities in these areas. Close monitoring of the spread of infection in these areas, including infectious disease control measures such as contact tracing, needs to be undertaken. In situations of extreme overcrowding and poor sanitation, evacuation of these camps must be considered. Currently, Doctors Without Borders is urging for the evacuation of refugee camps in Greece (65).

Language barriers also greatly limit the access of immigrants and refugees to public health messages (63)^.^ These populations must be empowered to take control of their health and engage with prevention strategies, by being provided accurate information in the appropriate languages. Given that refugees are often disadvantaged or vulnerable, they are less likely to trust governments or political systems. Thus, the involvement of stakeholders and leaders of these communities is critical to ensuring that preventative measures such as social distancing and handwashing are strictly adhered to whenever possible. Legal constraints decline refugees and immigrants' access to government welfare such as Centrelink (66). In such times of financial hardship, the livelihood of these populations is at risk of being severely compromised. To allow families to maintain food security, accommodation and health, governments should provide financial assistance, in recognition of these exceptional circumstances. Several countries have imposed strict border controls in response to the pandemic (67). This puts those who are seeking asylum at significant vulnerability and is against the spirit of international refugee law. Appropriate legal aid to asylum seekers should be provided by international humanitarian organizations. The long-term impact of COVID-19 on refugees and asylum seekers need further research.

### Impact on Justice-Involved Populations

The justice-involved population living in jails, prisons or custodial settings are particularly vulnerable in the COVID-19 era owing to the increased viral infection transmission risks due to crowded living conditions (68, 69), and relatively higher prevalence of specific medical conditions including poor cardiovascular disease profile, tuberculosis, sexually transmitted infection, substance abuse, and mental health disorder (70, 71). Jail inmates and prisoners have average to high cardiovascular disease (CVD) risk compared with community dwellers (72). Notably, individuals from lower socioeconomic backgrounds or belonging to ethnic or minority groups are inordinately incarcerated (73, 74). Moreover, history of incarceration is associated with CVD risk factors and poor prognosis (even death) from CVD (71). Therefore, targeted public health measures to minimize the transmission within this vulnerable population living in correctional facilities should be considered (75).

### Impact of COVID-19 on Remote Areas

People living in remote areas are more likely to be impacted by travel restrictions imposed by governments, as they need to travel long distances to access healthcare (76). This is particularly concerning for medical emergencies (31, 77). With the reorganization of health systems and repurposing of healthcare workers, it may be challenging to avail treatment at health facilities (29). Travel restrictions will also jeopardize ongoing care of chronic disease patients, who may not be able to access treatment locally.

Telemedicine facilities should be made available to enable the continued availability of healthcare to people living in remote areas (10, 78, 79). This has proven to be challenging as increased demand and inadequate staffing of internet providers have negatively impacted broadband services. Efforts must be taken to improve access to the internet, particularly for remote populations who are often unable to access healthcare by any other means. Deployment of mobile COVID-19 testing facilities should also be considered, to enable early detection and control of infection in rural and remote populations.

### Impact of COVID-19 on Chronic Diseases

The burden of COVID-19 has been severe for patients with chronic diseases such as obesity, cardiovascular diseases (heart disease and stroke), cancer, diabetes, chronic respiratory diseases, bone and joint disorders, genetic disorders, chronic neurological diseases (on immunosuppressive therapies (multiple sclerosis) and bulbar weakness (motor neurone disease) and mental disorders (29, 30). Patients with chronic disease are at increased risk of COVID-19 infection (80, 81). Mortality due to COVID-19 among chronic diseases could be substantial (81). Chronic disease patients with underlying risk factors such as age, obesity, lack of physical activity, tobacco use, poor nutrition and excessive alcohol consumption could be exposed to further risks due to COVID-19. Those with infection are at relatively higher risk of fatality (82, 83). Increased concerns about continuity of care, healthcare worker shortage, reorganization of health services, and limited access to testing and medical supplies have severely impacted patients with chronic diseases (83).

### Impact of COVID-19 on Patients With Cardiovascular Disease

Patients with underlying cardiovascular diseases, such as but not limited to–heart diseases, chronic stroke, obesity, and diabetes, are recognized to be at high risk of COVID-19 infection (29, 84–92). Substantially higher mortality risk has also been observed among critically ill COVID-19 patients. Acute cardiological manifestations of COVID-19 including heart failure, arrhythmia, left ventricular dysfunction and acute coronary syndromes have been observed (92). Indications on decreasing hospital presentations of acute cardiac events, presumably due to COVID-19 fear among patients, are concerning (93). Marked health inequities exist among individuals with cardiovascular diseases, diabetes and obesity across both low-income, middle income and high-income countries (94). The Prospective Urban Rural Epidemiologic (PURE) study found that the low-income and middle-income countries (LMICs) carry the highest burden of cardiovascular disease (95). Lower levels of education in LMICs are associated with increased CVD incidence rates and CVD-linked absolute case fatality rates (CFRs) despite better overall CVD risk factor profiles. However, these individuals have markedly poorer medical care apropos to management of diabetes and hypertension, and secondary prevention (95). Moreover, ethnicity and race linked disparities exist in CVD disease risk and associated deaths, e.g., the higher prevalence of CVD risk factors among Black patients than the Whites (96, 97). The impact of psychosocial (e.g., stress among patients with CVD) and environmental (pandemic) stressors, due to social distancing and quarantine measures, on cardiovascular health in general, and those with CVD in particular warrant further research.

### Impact of COVID-19 on Cancer Populations

Cancer patients bear a great financial burden, due to the significant expenses associated with treatment. This has a disproportionate impact on people of low socioeconomic status (SES) who are often unable to afford therapy. Nationwide data from Australia showed that cancer outcomes in patients were influenced by patients' post-codes, with those living in low socioeconomic areas having the lowest 5-year survival and highest mortality rates (98). Another analysis of people with metastatic breast cancer in the United States (US) found that uninsured people were more likely to refuse or delay treatment due to cost, compared to those who were insured (99). As resources in public hospitals become increasingly scarce, patients of low SES that do not have access to private healthcare may experience greater delays in treatment. In Australia, remote areas receive an undersupply of medical practitioners and diagnostic facilities, resulting in diagnostic delays, limited early detection, and significantly poorer cancer outcomes in rural populations. Moreover, these patients are often required to travel to metropolitan areas to receive treatment (100). The COVID-19 pandemic is likely to exacerbate these conditions, as travel bans and disruption of existing cancer services further limit access to treatment for these populations (7). The burden is worsened in developing countries such as India, where nearly 70% of the national population resides in rural areas and must travel to urban tertiary care centers for treatment (101, 102). Studies have also revealed racial disparities in access to cancer treatment worldwide (103, 104). Furthermore, inequalities between SES groups are also significant within developing countries. Although data from low-income countries is sparse, the available evidence indicates higher mortality rates among people with lower SES (105).

### Impact of COVID-19 on Chronic Neurological Patients

The reorganization of the healthcare system, as well as travel restrictions, has made it challenging to maintain ongoing care of patients with chronic neurological conditions especially those on immunosuppressive treatment regimen (e.g., multiple sclerosis) or with bulbar weakness (e.g., motor neurone disease) (30). While telemedicine is being implemented as a substitute for in-person consultations, many patients, due to limited literacy and access to appropriate technology may be unable to access services (10). Doctors have reported a sudden decrease in the number of patients presenting to hospitals with acute neurological events such as stroke, likely due to fear of contracting COVID-19 infection (31, 93, 106). This is problematic as the delay in reperfusion therapy may have fatal consequences (107–109), especially to patients with underlying chronic neurological disease (30). Likewise, there are also concerns related to the post-ponement of elective surgeries, therefore, the impact of delayed surgery on long-term morbidity needs further study. Health strategies to minimize the impact of the pandemic on patients with neurological conditions should include considerations of individual comorbidities or health profile as well as the socioeconomic variables associated with health. Patients who might be at high risk of an acute flare-up (such as transient ischemic attack or acute stroke) should be monitored using telemedicine and if necessary, be brought for the emergent procedure (30, 31). In the wake of COVID-19, certain diagnostic and treatment workups need to be reconsidered to limit infection exposure to patients and healthcare workers.

### Impact on Mental Health

Before the COVID-19, mental health has been a subject of major concern due to rising numbers around the world (110). In the pre-COVID era, worldwide, 800 000 people died due to suicide every year; it was a second leading cause of death among youth (15–29-year olds) globally (111). The major burden of suicides occurs in LMICs accounting for 79% of all suicides recorded globally in 2016. For example, in Australia alone, on average 8 Australians commit suicide every day, with suicide rates in 2015 amongst Aboriginal and Torres Strait Islander people (5.2%) were more than double the national rate (1.8%) (112). In addition to the personal and family suffering as well as damage to the community mental illness causes, the costs to the economy are gigantic (113).

In the COVID-19 era, social distancing, increasing joblessness and limited access to mental health services, mental health burden is bound to aggravate during and beyond the pandemic (110). Several surveys on general public have reported increase in COVID-19 related depression, anxiety, and stress owing to the psychosocial stressors (114–117). Among healthcare workers, clinical symptoms of depression, anxiety, insomnia, and distress have been reported with a prevalence rate higher than the general public (27). Lack of social support and communication, a lack of disaster training and maladaptive coping strategies increase the risk of negative psychological or mental outcomes (27, 118). To reduce adverse psychological outcomes among frontlines healthcare workers, targeted preventive and mitigation measures including stress coping strategies are recommended (118, 119).

Increasing loneliness, economic downturn, and stress invoked by living through a crisis place the entire population at high risk of psychological disorders such as depression, anxiety and substance abuse (120, 121). Zhang et al.'s study on health and well-being of normal adults after 1 month of confinement in China showed worse mental and physical health distress and life satisfaction among adults who did not work in the outbreak (122). This study gives insights on developing targeted interventions to limit the negative impact on health and well-being of specific groups who might benefit from appropriate intervention including targeted treatment and social assistance.

Social restriction and quarantine imposed by various governments during COVID-19 will have a psychological and psychiatric impact (123, 124). Moreover, mental health consequences are likely to be more severe in socially vulnerable populations that are experiencing the greatest challenges during the pandemic (125, 126). Provision of remote psychiatric or psychological assistance through telemedicine should be considered (10). The vulnerable communities would need targeted interventions to help them cope with loneliness, fear, stigma and acute distress (127). Mental health screening programs to identify those at higher risk of suicide could be explored. Programs that have shown efficacy in improving psychological and mental health during previous epidemics such as the Zika virus outbreak could be used as a foundation to develop COVID-19 specific interventions (128). Population-level programs to monitor behavioral, interpersonal and psychological reactions to the COVID-19 could be considered to identify populations or communities at greatest risk.

### Impact of COVID 19 on Critically Ill Patients and Their Families

The COVID-19 patients who are critically ill, spend their last few days in ICU separated from their loved ones. The lack of PPE and strict ICU isolation protocols led to the death of most patients in ICU alone and away from their close family (129). The health care personnel who communicate to the close family members, experience extreme anguish, and this has a lasting psychological impact and moral injury on the health care workers. In several countries, visits to critically ill and funerals have been banned due to the high risk of contagion (129). This in several cultures would lead to intense pain and suffering to the family as it could be against their respective culture and tradition. Nobody wishes to die alone, but the fact that many patients are dying alone without being able to meet or communicate with their family one last time. Some countries are allowing family viewing of the deceased under strict infection control measures (130). Just like how telehealth and virtual meetings are becoming the new normal, we may have to use telecommunication for the patients to be able to communicate with their families remotely. This may not be evidence-based medicine, that we so wish to practice, but maybe considered part of compassionate care provided to the dying, as well as to their friends and family.

## Discussion

**Figure 1:**
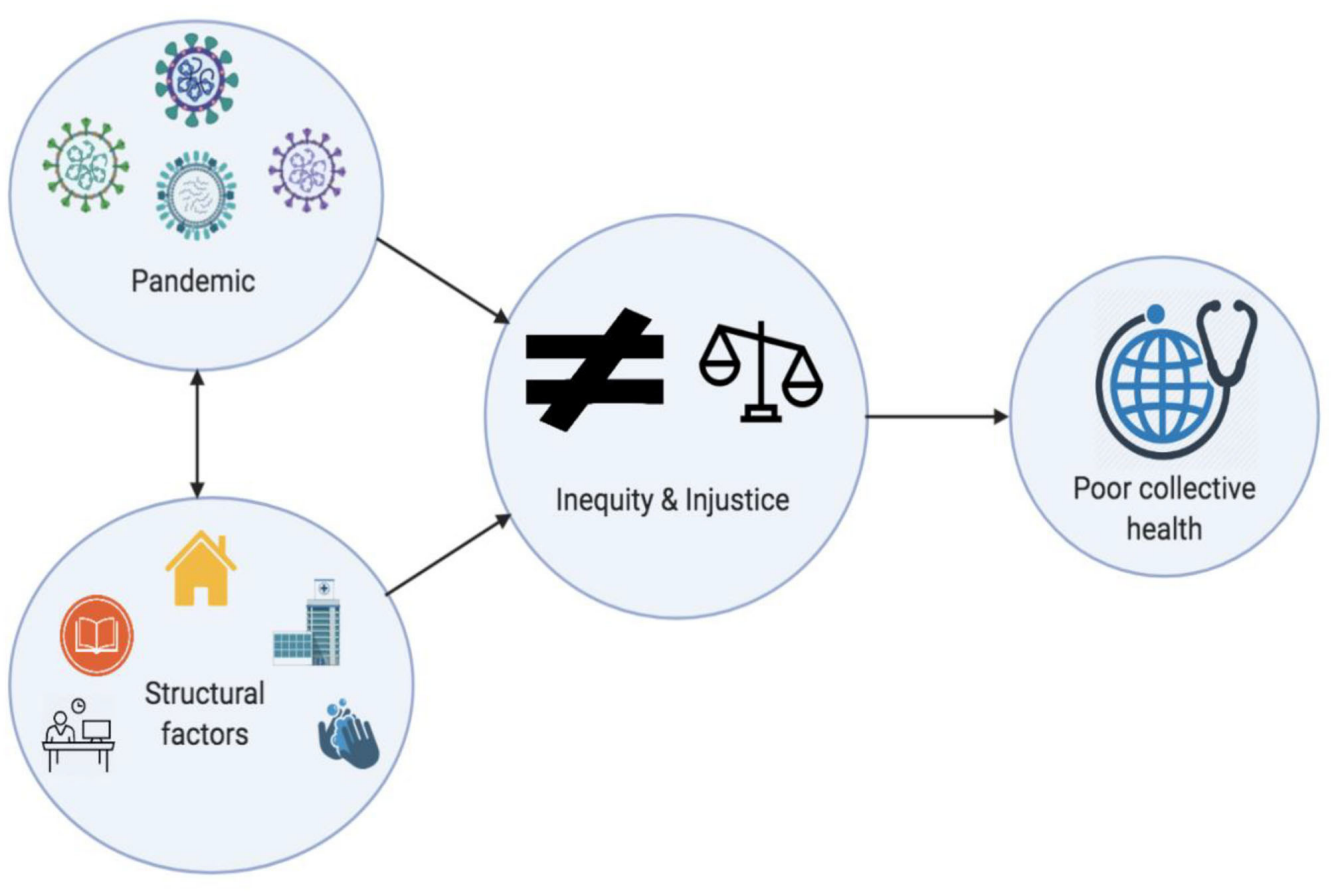
Schematic showing how pandemics potentiate structural factors to cause compounded negative impact on equity and justice.

Prolonged public health or humanitarian crises can act as a catalyst to realign civilizational priorities with a focus on health, equity and justice. COVID-19 has led to an unprecedented surge in fear, dismay and disbelief (22, 52, 121, 128). Pandemics have a disproportionate impact on the health and socioeconomic status of people from vulnerable backgrounds (Figure 1). These communities face exacerbated inequity and injustice (131). Healthcare workers, homeless, elderly, people of lower socioeconomic backgrounds, those from ethnic or minority groups, immigrants and/or refugees, justice-involved populations (incarcerated), people with disabilities, those living in remote areas, with disabilities and with chronic conditions are experiencing a disproportionate burden of the COVID-19. Increasing media reports are highlighting the palpable inequity and injustice that are currently being experienced by the community, more so by those who come from vulnerable backgrounds. These vulnerabilities are further exacerbated in under-resourced settings. The impact of the disease has ramifications well-beyond those who have been infected and those who die from the infection. The pandemic of this scale and geographical breadth have raised serious concerns about the capacity of our health systems to cope (132). On a community level, we are witnessing a breakdown of existing infrastructures causing challenges in access and compliance. History tells us when aspects of equity and justice are neglected, the systems either have to be restructured or it will be forced to reboot by default (133).

Recent statements by the Director of National Institute of Allergy and Infectious Diseases in the United States of America, Dr Anthony Fauci, about the possibility “that Americans could eventually carry around certificates of immunity to the coronavirus once proper testing is widespread enough” raise concerns (134). How this will impact vulnerable communities remain to be seen. This along with the idea of creating an “immunity registry” are also concerning. There are concerns that such a registry could be misused to profile those from vulnerable communities leading to further marginalization. The announcement by the current administration in the United States, to put a halt on the funding to the World Health Organization (WHO), will severely impact the international coordination efforts to mitigate COVID-19 outbreak as the United States is a major funder (135). COVID-19 has invoked a raging debate on the urgent need to revisit commitments by individual governments toward health equity and justice (136). In this vein, models of universal healthcare that have been successfully implemented in some countries may act as templates for relevant governments to prioritize health and justice for their populations (137). Contemporary models to combat inequities in access to care and protect vulnerable communities give hope to build sustainable universal healthcare infrastructure. The *Ayushman Bharat Pradhan Mantri Jan Arogya Yojana* scheme introduced by the Indian government provides free health coverage to 500 million of the most disadvantaged members of the Indian population and its implementation has been promising (138). Brazil's national health system–*Sistema Único de Saúde* (SUS)–provides financial support to approximately 70% of its population (139). Spain also has a public health system which assures universal coverage for all Spanish nationals regardless of economic background (140). These systems provide “safety nets” to the most vulnerable communities in hours of crisis. A more equitable allocation of funds for the improvement of primary healthcare in all countries across the globe is the need of the hour (141). The doctor-patient ratio has to be improved to provide a uniform distribution of healthcare to remote locations and lower socioeconomic status (142).

Ongoing management of patients with chronic disease is essential to minimizing the progression of the disease and should be ensured (29). Doctors are recommended to utilize telemedicine to reach out to patients virtually when possible and reduce in-person visits to the clinic (10). To facilitate this, we urge governments to reimburse all healthcare providers for teleconsultations. Patients should continue receiving treatment, unless the risks of doing so outweigh the benefits, as per the judgement of the responsible clinicians. If treatment cannot be administered remotely, patients should be encouraged to present to hospitals for treatment, and all necessary precautions must be taken to minimize the risk of COVID-19 infection transmission. For patients who do not have access to a private vehicle, provision of transportation to and from the hospital could be organized for patients with limited physical mobility. Early detection of chronic diseases is crucial to prevent fatal progression. Thus, we recommend the continuation of existing screening activities (e.g., breast cancer screening program), with strict adherence to PPE and handwashing guidelines. Patients with acute cardiac and/or neurological symptoms should be encouraged to access treatment as the absence or lack of treatment could have a devastating impact on individuals (31).

Contracting COVID-19 infection could be fatal for patients with chronic disease particularly those with severely compromised immune systems and CVD risk factors including but not limited to obesity and advanced age (29). We recommend regular telemonitoring of all patients for COVID-19 symptoms, such that immediate action can be taken for suspected infection. Chronic disease wards should be divided into COVID-19 positive and COVID-19 negative wards. Patients with a suspected infection should undergo diagnostic testing, and those who test positive should be monitored for progression of symptoms. If their condition deteriorates, we recommend direct routing of these patients to COVID-19 positive chronic disease ward, to circumvent exposure to the emergency department. Critically ill patients and their loved ones should be given special consideration on compassionate grounds so that the families and the ICU staff could have closure and potential adverse psychological impact on healthcare staff (due to moral injury) and families could be thwarted.

In conclusion, unprecedented times deserve unprecedented measures. The social and economic determinants of health mediate the impact of crises such as a pandemic (41–45, 143). Factors related to individual socioeconomic status, underlying morbidity and external factors such as diminishing access to healthcare and justice systems could deepen the inequity and injustice divide during and beyond the COVID-19 era (13, 131, 141). Factors such as universal health care access, provision of education for all, protection from disasters including caused by pandemics and climate change, justice to all, and equality in opportunity affect how communities and nations respond and cope with the crisis (51, 131). For us to have a comprehensive approach to be able to recoil back into a functioning society, efforts to address these determinants are important. Digital technologies such as big-data analytics and artificial intelligence could be leveraged in surveillance and care of people from vulnerable communities during and beyond-COVID-19 (79). Furthermore, individual governments should allocate dedicated funding to support ongoing research and development and public health surveillance of current and long-term impact of COVID-19 on vulnerable populations. Without a holistic approach to building sustainable and inclusive systems that address health inequity and injustice, we will continue to be vulnerable to pandemics such as one that we face now, and those that may occur in future (144). It is also important that COVID-19 mitigation strategies should not stigmatize or marginalize vulnerable communities (9). Identifying vulnerable members of the community and those at high-risk should be an integral part of pandemic public health response strategies. We urge governments to take a proactive approach toward realignment of national efforts on creating sustainable planetary health, justice and environmental systems–one that could protect our generations in face of a pandemic or prolonged crisis (12, 136, 145).

## Author's Note

The COVID-19 pandemic is causing an unprecedented public health crisis impacting healthcare systems, healthcare workers, and communities. The COVID-19 Pandemic Health System REsilience PROGRAM (REPROGRAM) consortium is formed to champion the safety of healthcare workers, policy development, and advocacy for global pandemic preparedness and action. This paper could only consider developments until April 30, 2020, at the time of manuscript writing and submission; however, some updates were considered and added during the revision of the manuscript in October–November 2020.

## Author Contributions

SBh devised the project, the main conceptual ideas and proof outline, and coordinated the writing and editing of the manuscript. SBh and AR wrote the first draft of the manuscript. SBh encouraged AR to investigate and supervised the findings of this work. All authors discussed the results and recommendations and contributed to the final manuscript.

## Conflict of Interest

The authors declare that the research was conducted in the absence of any commercial or financial relationships that could be construed as a potential conflict of interest.
